# Social support and medication adherence in type 2 diabetes: unraveling the sequential mediating pathways of empowerment and health literacy

**DOI:** 10.3389/fpubh.2026.1783412

**Published:** 2026-07-08

**Authors:** Xiaoxia Meng, Li Xie, Xianfeng Zhou, Min Zhang, Dan Zhang

**Affiliations:** 1 School of Medicine and Health Management, Guizhou Medical University, Guiyang, China; 2 Department of Endocrinology and Metabolism, The Affiliated Hospital of Guizhou Medical University, Guiyang, China; 3 School of Pharmaceutical Science, Guizhou Medical University, Guiyang, China

**Keywords:** empowerment, health literacy, medication adherence, social support, type 2 diabetes

## Abstract

**Objective:**

Medication adherence is critical for effective blood glucose control and reducing complication risks in patients with Type 2 Diabetes Mellitus (T2DM). However, improving it remains challenging in clinical practice and disease management. This study examined the relationships among social support, empowerment, health literacy, and medication adherence in T2DM patients to explore the multi-path associations underlying medication adherence.

**Methods:**

Social support, empowerment, health literacy, and medication adherence constituted the theoretical model. A cross-sectional survey was conducted using convenience sampling at a Grade III Class A hospital in Guiyang City, China. Univariate analyses were performed using nonparametric rank-sum tests. Structural equation modeling (SEM) was applied to test the hypothesized model, and a bootstrap was performed to examine mediation effects.

**Results:**

A total of 261 T2DM patients were included, with a median medication adherence score of 6.75 (IQR 4.50, 7.63), indicating moderate adherence on the MMAS-8. In univariate analysis, educational level showed the strongest association with medication adherence (H = 67.452, *p <* 0.001). Social support was positively associated with medication adherence through both direct (53.5%) and indirect (46.5%) pathways. The indicators for affectionate support (*β* = 0.77) and emotional information support (*β* = 0.72) had the highest standardized factor loadings. Health literacy acted as a significant mediator (effect = 0.256, 95% CI: 0.133–0.460, 31.7%), and a small but significant sequential mediation pathway was observed (effect = 0.062, 95% CI: 0.018–0.147, 7.7%).

**Conclusion:**

In this hospitalized sample of Chinese T2DM patients, empowerment and health literacy sequentially mediated the association between social support and medication adherence. These findings suggest that social support and health literacy may be relevant targets for future intervention research, although longitudinal studies are needed to establish their temporal and causal roles.

## 1 Introduction

According to the 11th edition of the World Diabetes Map released by the International Diabetes Federation (IDF), 589 million people worldwide are living with diabetes (1), and China currently bears the world’s largest population of individuals with type 2 diabetes, with an adult prevalence of 11.2% (2, 3). Pharmacological treatment is a crucial component of the holistic management of Type 2 Diabetes Mellitus (T2DM), and medication adherence is vital for regulating blood glucose levels and preventing diabetic complications (4). However, numerous studies have indicated that medication adherence among diabetic patients is far from optimal (5, 6). According to a survey in China, 61.9% of inpatients with T2DM had poor medication adherence (7).

In recent years, the associations of social support, health literacy, and diabetes empowerment with medication adherence in patients with T2DM has gradually attracted attention in the research field. Social support refers to the emotional, informational, and practical support that patients obtain from their social networks and is associated with medication adherence behaviors (8). Among hospitalized patients, those with low social support have 2.48-fold higher odds of nonadherence to antidiabetic medications compared with those with high social support (7). The relationship between social support and medication adherence may involve multiple pathways. Social support is not only directly associated with medication adherence but also may show an indirect association through enhanced disease acceptance (9). Another study showed that social support was indirectly associated with medication adherence through self-efficacy (10). Understanding the relationship between social support and medication adherence is of great significance for designing intervention measures, especially for patients living alone or with limited social support networks. Recognizing the multidimensional nature of social support and its correlates holds important clinical implications for improving medication adherence in patients with diabetes.

Diabetes empowerment is a specific application of psychological empowerment in chronic disease management. It refers to a practical model that positions patients at the center of their own health management by strengthening their disease management skills, confidence, and intrinsic motivation, thereby enabling them to make treatment decisions independently and ultimately improving their quality of life (11, 12). Diabetes empowerment has been linked to sustained self-care behaviors and blood glucose monitoring, favorable HbA1c outcomes (13–17), and better medication adherence (18).

Diabetes health literacy refers to the requisite skills and competencies that enable patients to acquire, comprehend, evaluate, and convey diabetes-related information in both clinical settings and daily life, as well as to use such information to improve their health (19, 20). Evidence has demonstrated a strong correlation between adequate diabetic health literacy and improved glycemic control (21, 22). A systematic review indicated that the majority of studies demonstrated a significant positive association between health literacy and medication adherence (23). Limited health literacy is associated with poor medication adherence and with adverse outcomes, including high rates of emergency admissions, prolonged treatment and hospital stays, and increased mortality (24).

Although the independent associations of social support, diabetes empowerment, and health literacy with medication adherence are well established in the literature, the relationship among the four constructs is still unclear. Existing mediation analyses in this field have focused almost exclusively on isolated single-mediator models. For example, several studies examined the mediating role of health literacy alone (25, 26), while another study examined the mediating role of empowerment in the relationship between patient-centered care and medication adherence (18). They fail to situate social support, diabetes empowerment, and health literacy within an integrated framework, making it impossible to unravel the sequential pathways among these constructs. Moreover, previous studies rely heavily on empirical observations without grounding in a unifying macro-level behavioral theory, leaving the psychological mechanisms associated with medication adherence insufficiently articulated.

To address these research gaps, we proposed a theory-based sequential mediation model to explore how social support is associated with medication adherence in patients with T2DM. The model takes Bandura’s Social Cognitive Theory (SCT) (27) as the main framework, integrating Nutbeam’s Hierarchical Model of Health Literacy (28) and Diabetes Empowerment Theory (11). SCT posits triadic reciprocal determinism, which means that an individual’s health behavior is shaped by the dynamic, reciprocal interaction among environmental, personal cognitive, and behavioral factors (27). Personal cognitive factors, especially self-efficacy and intrinsic motivation, serve as the key intermediary between environmental influences and sustained behavior change. In our research model, social support serves as an environmental factor, empowerment and health literacy as personal cognitive factors, and medication adherence as the behavioral factor. Empowerment and health literacy act as the intermediary between social support and medication adherence.

**Figure 1:**
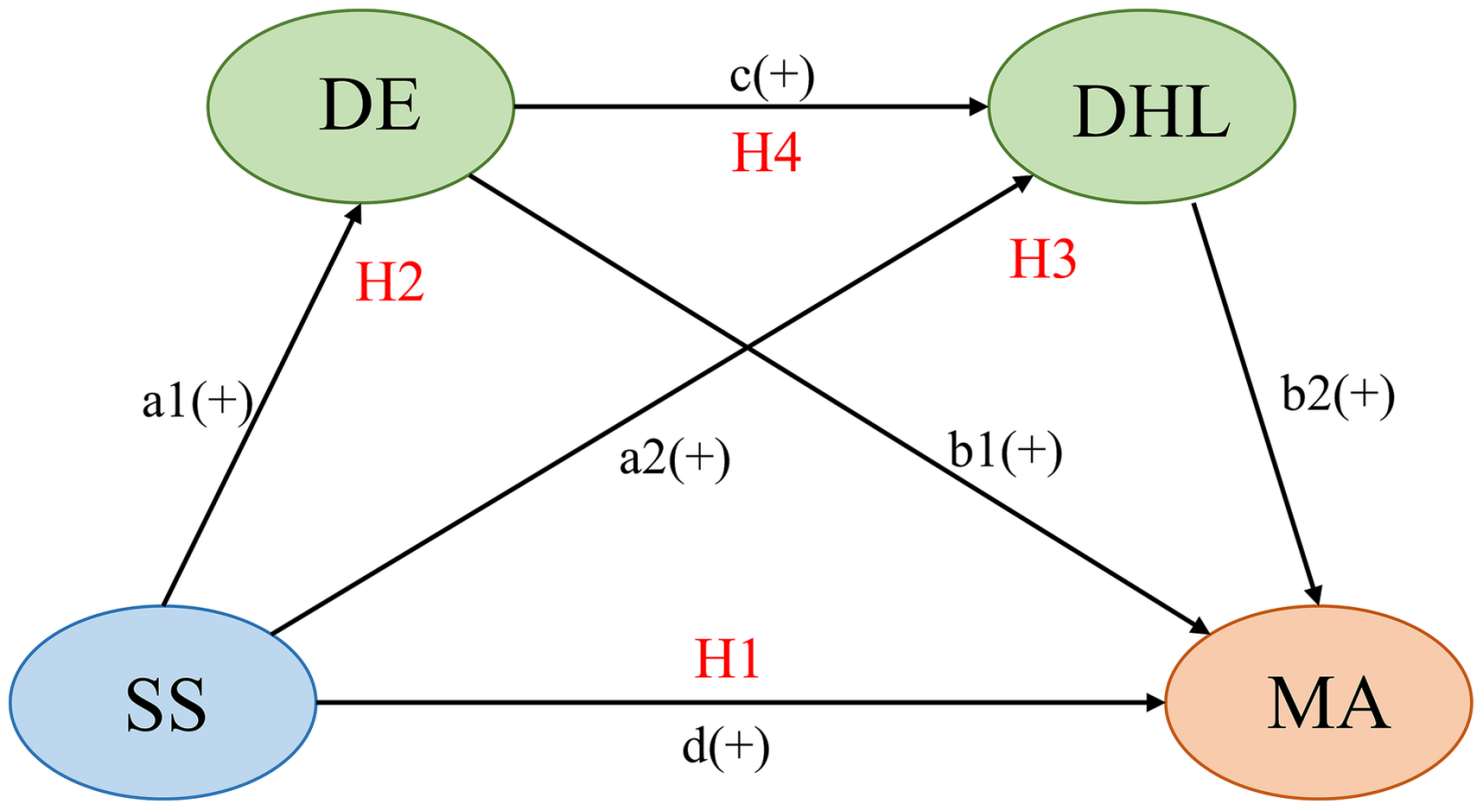
Conceptual framework. (1) SS, Social Support; DE, Diabetes Empowerment; DHL, Diabetes Health Literacy; MA, Medication Adherence. The purple construct represents exogenous construct; the green constructs represent mediating constructs; the orange construct represents outcome construct. (2) All paths are hypothesized to be positive (+). (3) Hypothesis-path correspondence: H1 (direct effect): SS → MA (path d). H2 (parallel mediation 1): SS → DE → MA (paths a1 × b1). H3 (parallel mediation 2): SS → DHL → MA (paths a2 × b2). H4 (sequential mediation): SS → DE → DHL → MA (paths a1 × c × b2).

Regarding the order of the two mediators, the pathway from diabetes empowerment to health literacy was established according to SCT’s motivation priority principle and Nutbeam’s Hierarchical Model of Health Literacy. Nutbeam’s model conceptualizes health literacy as three progressive and hierarchical levels: functional health literacy (basic reading, writing, and numeracy skills), interactive health literacy (skills to communicate with others and obtain health information), and critical health literacy (the ability to critically evaluate health information and make autonomous health decisions) (28). The latter two levels are closely linked to sustained and long-term health behavior change. The activation and effective application of these higher-order skills, however, depend fundamentally on an individual’s willingness and intrinsic motivation to actively participate in health management, which is precisely the core connotation of diabetes empowerment. Patients who are not empowered are unlikely to proactively seek, evaluate, or apply health information to achieve meaningful, sustained improvements in health literacy, even if they possess basic functional literacy skills. Two longitudinal studies further support this direction, demonstrating that baseline empowerment significantly predicts subsequent improvements in health literacy (29, 30). Conversely, the reverse sequence, which links health literacy to diabetes empowerment, lacks theoretical support. According to SCT, cognitive skills do not automatically generate self-efficacy or intrinsic motivation, and motivation must precede the acquisition and application of such skills. Health literacy is essentially a set of cognitive skills that cannot directly give rise to diabetes empowerment. Similarly, Nutbeam’ s hierarchical model holds that advanced health literacy cannot be developed or sustained without the active engagement enabled by empowerment (28). Finally, consistent with SCT’s core premise that personal cognitive factors directly shape behavior (27), health literacy serves as the proximal mediator linking psychological status to medication adherence. Patients with higher diabetes health literacy can more accurately understand medication regimens, follow clinical guidance, communicate effectively with providers to resolve treatment-related barriers, and thus maintain consistent, long-term medication adherence. In summary, we hypothesize a sequential pathway in which social support first relates to diabetes empowerment, which facilitates health literacy, ultimately contributing to medication adherence. This study proposes the following four hypotheses (Figure 1).

*Hypothesis 1*: Social support is positively associated with medication adherence in T2DM patients.

*Hypothesis 2*: Diabetes empowerment mediates the relationship between social support and medication adherence.

*Hypothesis 3*: Diabetes health literacy mediates the relationship between social support and medication adherence.

*Hypothesis 4*: Diabetes empowerment and health literacy sequentially mediate the relationship between social support and medication adherence.

To test the above hypotheses, this cross-sectional study recruited hospitalised patients with T2DM from the Endocrinology Department of a tertiary hospital (Grade III, Class A) in Guiyang, China. Structural equation modelling (SEM) was applied to examine the direct and mediating pathways among the constructs. The study aimed to explore the multi-path associations underlying medication adherence in T2DM patients, thereby providing insights that may inform future intervention strategies for clinicians and public health professionals.

## 2 Materials and methods

### 2.1 Participants and data collection

A convenience sample of newly admitted in patients with T2DM was recruited from the Department of Endocrinology of a tertiary hospital (Grade III, Class A) in Guiyang, China, between April and September 2025. This 6-month data collection period was selected for two main reasons. For one reason, given that the typical hospital stay for patients with T2DM in this unit is 7–10 days, a new cohort of patients is admitted approximately every 10 days. A 6-month recruitment period was necessary to gather a sufficient sample size representing patients admitted across different weeks and months, and to minimize bias from temporary changes in patient characteristics. For another, the period from April to September in Guiyang is characterized by a stable, mild climate, which contributes to a relatively consistent volume of diabetes-related admissions. This timeframe also avoids the peak of respiratory infections in winter, a factor known to cause notable blood glucose fluctuations in patients with T2DM (31) and to potentially affect their medication adherence.

The inclusion criteria were as follows: (1) a diabetes duration of at least 6 months; (2) age 18 years or older; (3) normal cognitive function and intact communication ability; and (4) provision of informed consent and willingness to participate. The exclusion criteria included: (1) severe physical comorbidities, severe cognitive impairment, or psychiatric disorders; (2) impaired verbal communication ability; and (3) gestational diabetes mellitus. The study was approved by the Ethics Committee of Guizhou Medical University under the approval number 2021–203.

Sample size was determined based on established guidelines for covariance-based structural equation modeling (CB-SEM). The fundamental sample-to-free-parameters ratio principle was applied. The model contained 36 free parameters, corresponding to a minimum requirement of 180 participants (5:1 ratio) and a recommended 360 participants (10:1 ratio) (32). In addition, the widely accepted minimum threshold of 200 participants for stable parameter estimation and model convergence was adopted (33). Moreover, MacCallum’s RMSEA-based power analysis (*α* = 0.05, power = 0.80, df = 100) indicated a required sample size of 175 (34). We distributed 265 questionnaires and obtained 261 valid responses, which exceeded all minimum criteria and provided sufficient statistical power for reliable hypothesis testing.

Before the survey, all researchers underwent uniform training. Participants were informed of the primary purpose of the study, and face-to-face surveys were conducted after they provided informed consent. Questionnaires were collected immediately upon completion. The data were imported into the statistical analysis program after being independently verified for accuracy by two researchers.

### 2.2 Measurements

#### 2.2.1 Demographics

The demographic variables were developed based on the research objectives and literature review. These variables included gender, age, marital status, educational level, monthly income, occupational category, disease duration, and the presence of diabetes-related complications.

#### 2.2.2 Social support

Social support was evaluated using the Chinese version of the Medical Outcomes Study Social Support Survey (MOS-SSS-C). The original Medical Outcomes Study Social Support Survey (MOS-SSS) was developed by Sherbourne and Stewart in 1991 and was primarily used to assess the level of social support in patients with chronic diseases (35). The Chinese version was revised by Yu et al. (36) at the Chinese University of Hong Kong in 2004. It contains a total of 19 items, covering four dimensions: tangible support (4 items), affectionate support (3 items), social interaction support (4 items), and emotional information support (8 items). Each item is rated on a 5-point Likert scale, ranging from 1 (“none of the time”) to 5 (“all of the time”), with a total score range of 19–95. A higher score indicates greater social support. Wang et al. (37) confirmed that the scale has good reliability and validity in a study of patients with coronary heart disease. The Cronbach’s *α* coefficient in this study was 0.925, further indicating its high internal consistency.

#### 2.2.3 Diabetes empowerment

Diabetes empowerment was evaluated using the Diabetes Empowerment Scale-Short Form (DES-SF). This scale was created by Anderson et al. (38) and subsequently translated into Chinese by Hu et al. (39). The scale comprises 8 items, each rated on a 5-point Likert scale ranging from 1 (“strongly disagree”) to 5 (“strongly agree”). In the Chinese validation study, the Cronbach’s *α* coefficient of the scale was 0.848 (39). In this study, Cronbach’s α was 0.840.

#### 2.2.4 Diabetes health literacy

Diabetes health literacy was evaluated using the Diabetes Health Literacy Scale (DHLS), which was developed and validated by Lee et al. (20). This scale comprises 14 items across three dimensions: informational health literacy, numeracy health literacy, and communicative health literacy. Each item is rated on a 5-point Likert scale, ranging from 1 (“strongly disagree”) to 5 (“strongly agree”). The overall score ranges from 14 to 70, with higher scores indicating better health literacy. The Cronbach’s *α* in this study was 0.909.

#### 2.2.5 Medication adherence

The Morisky Medication Adherence scale (MMAS-8) is a widely utilized self-report instrument specifically designed to assess medication adherence (40). It has been translated into multiple languages and applied across diverse research contexts. The Chinese version of the MMAS-8 was translated and adapted by Wang et al. (41). For items 1 to 7, a “no” response is scored as 1 and a “yes” as 0, except for item 5, where the scoring is reversed (“yes” = 1, “no” = 0). Item 8 is scored as follows: “Never” = 1, “Rarely” = 0.75, “Sometimes” = 0.50, “Usually” = 0.25, and “All the time” = 0. The total MMAS-8 score ranges from 0 to 8, with higher scores indicating better adherence. Adherence levels are categorized as low (<6), medium (6 to <8), and high (8). In the Chinese validation study, the scale demonstrated excellent test–retest reliability (ICC = 0.80) and moderate internal consistency (Cronbach’s *α* = 0.65) (41). In this study, Cronbach’s *ɑ* was 0.776.

### 2.3 Statistical analysis

This study used SPSS 26.0 for statistical description and analysis. All questionnaires were checked for completeness and logical consistency immediately upon collection. There were no missing values, so all data were directly included in the statistical analyses. Categorical variables were presented as composition ratios (%). Scores for social support, diabetes empowerment, diabetes health literacy, and medication adherence were first assessed for normality using the Shapiro–Wilk test (*α* = 0.05). Since the distributions were non-normal, descriptive statistics were reported as medians (M) and interquartile range (P25, P75). Common method variance (CMV) was assessed using Harman’s single-factor test and Common Latent Factor (CLF) model. Univariate analyses were performed using nonparametric rank-sum tests, and associations between variables were quantified with Spearman’s correlation coefficients. Discriminant validity was assessed by comparing the square root of the average variance extracted (AVE) for each construct with its correlations with other constructs. Structural equation modeling (SEM) was performed using AMOS 28.0 software with maximum likelihood (ML) estimation. Multi-group structural equation modeling was used to conduct measurement invariance testing, to confirm the measurement equivalence of the scale across groups with different educational levels. Mediation effects were examined using the Bootstrap procedure with 5,000 resamples (42). A mediation effect was deemed statistically significant if the 95% confidence interval (CI) did not contain zero. To control for potential confounding, we conducted a robustness test by including age, educational level, monthly income, and diabetes complications as exogenous covariates regressed on all endogenous latent variables to estimate adjusted net associations and mediating effects. All statistical tests were two-tailed, and a *p* < 0.05 was considered statistically significant.

## 3 Results

### 3.1 Demographic and clinical characteristics of the participants

As indicated in Table 1, this study included 261 patients with T2DM, of whom 143 (54.8%) were males and 118 (45.2%) were females. The largest age group was 60 to 74 years (*n =* 111, 42.5%), followed by 45 to 59 years (*n =* 83, 31.8%). Most patients were married (*n =* 216, 82.8%). Regarding educational level, the largest subgroup was primary school or lower (*n =* 76, 29.1%). In terms of monthly income, 101 patients (38.7%) reported 3,000 yuan or below, with the remaining income groups each accounting for a relatively similar proportion. Most patients were retirees (*n =* 91, 34.9%), followed by employees of enterprise, institution, and government organs (*n =* 55, 21.1%) as well as workers and farmers (*n =* 54, 20.7%). Regarding disease duration, 73 patients (28.0%) had a duration of 5 years or below, while an equal number of patients (*n =* 56, 21.5% for each group) had durations of 5 to 10 years and 10 to 15 years, respectively. In addition, 174 patients (66.7%) had diabetes-related complications, while 87 patients (33.3%) had none.

**Table 1:** Sample characteristics and medication adherence score comparison in type 2 diabetes patients (*N =* 261).

Characteristics		*N* (%)	Medication adherence		
			Score [M (P25, P75)]	Statistics	*p*-value
Gender	Male	143 (54.8%)	6.75 (4.75, 7.75)	Z = −2.736	**
	Female	118 (45.2%)	5.75 (3.50, 7.00)		
Age	18–44	30 (11.5%)	7.75 (6.50, 8.00)	H = 17.537	***
	45–59	83 (31.8%)	6.25 (4.50, 7.75)		
	60–74	111 (42.5%)	6.50 (4.00, 7.00)		
	≥75	37 (14.2%)	5.75 (2.75, 6.75)		
Marital status	Unmarried	8 (3.1%)	7.88 (7.19, 8.00)	H = 13.809	**
	Married	216 (82.8%)	6.75 (4.50,7.69)		
	Widowed	27 (10.3%)	5.25 (2.75, 6.75)		
	Divorced	10 (3.8%)	5.13 (2.81, 7.13)		
Educational level	Primary school and below	76 (29.1%)	4.36 (2.50, 5.75)	H = 67.452	***
	Junior high school	47 (18.0%)	6.00 (4.75, 7.00)		
	Senior high school and secondary vocational school	50 (19.2%)	6.75 (5.75, 7.81)		
	Junior college	35 (13.4%)	7.00 (5.75, 7.75)		
	Undergraduate	49 (18.8%)	7.00 (6.75, 8.00)		
	Master’s degree and above	4 (1.5%)	7.25 (6.56, 7.94)		
Monthly income	3,000 and below	101 (38.7%)	4.75 (2.75, 6.86)	H = 37.792	***
	3,001–5,000	61 (23.4%)	6.75 (4.75, 7.00)		
	5,001–8,000	52 (19.9%)	6.75 (4.75, 7.94)		
	8,001 and above	47 (18.0%)	7.00 (6.75, 7.75)		
Occupational Category	Enterprise, institution, and government organ employees	55 (21.1%)	6.75 (5.75, 7.75)	H = 20.065	***
	Workers and farmers	54 (20.7%)	5.50 (2.75, 6.81)		
	Self-employed	17 (6.5%)	5.75 (3.50, 7.75)		
	Retirees	91 (34.9%)	6.75 (4.50, 7.50)		
	Other	44 (16.9%)	5.75 (4.06, 7.75)		
Disease duration	≤5 years	73 (28.0%)	6.75 (4.50, 7.75)	H = 10.115	*
	5–10 years	56 (21.5%)	6.63 (4.00, 7.56)		
	10–15 years	56 (21.5%)	5.75 (4.50, 7.00)		
	15–20 years	36 (13.8%)	6.75 (5.75, 7.75)		
	>20 years	40 (15.3%)	5.75 (2.81, 6.75)		
Diabetes-related complication	Yes (≥1 complications)	174 (66.7%)	6.00 (4.50, 7.00)	Z = −3.102	**
	No	87 (33.3%)	6.75 (5.50, 8.00)		

*p < 0.05; **p < 0.01; ***p < 0.001; Medication adherence was measured using the Morisky Medication Adherence Scale-8 (MMAS-8). MMAS^®^ 2006 used with permission www.moriskyscale.com.

### 3.2 Comparison of medication adherence scores by characteristics

Univariate analyses were performed using nonparametric rank-sum tests. As shown in Table 1, the results indicated statistically significant differences in medication adherence scores among patients with T2DM across gender (Z = −2.736, *p <* 0.01), age (H = 17.537, *p <* 0.001), marital status (H = 13.809, *p <* 0.01), educational level (H = 67.452, *p <* 0.001), monthly income (H = 37.792, *p <* 0.001), occupation category (H = 20.065, *p <* 0.001), disease duration (H = 10.115, *p <* 0.05), and diabetes-related complications (Z = −3.102, *p <* 0.01). The results indicated that education level was the demographic factor most strongly associated with medication adherence.

### 3.3 Scores and correlation analysis of constructs

Table 2 presents the construct scores and Spearman correlation coefficients for social support, diabetes empowerment, and diabetes health literacy among the 261 T2DM patients. The median scores (interquartile range) were 71.00 (59.50, 80.00) for social support, 30.00 (26.00, 35.00) for empowerment, 52.00 (39.00, 60.00) for health literacy, and 6.75 (4.50, 7.63) for medication adherence. All constructs were significantly positively correlated (*p <* 0.01).

**Table 2:** Construct scores and Spearman’s correlation coefficients between constructs (*N =* 261).

Constructs	Score [Md (P25, P75)]	Constructs			
		SS	DE	DHL	MA
SS	71.00 (59.50, 80.00)	1			
DE	30.00 (26.00, 35.00)	0.509**	1		
DHL	52.00 (39.00, 60.00)	0.604**	0.503**	1	
MA	6.75 (4.50–7.63)	0.692**	0.563**	0.740**	1

**p < 0.01; SS, Social Support; DE, Diabetes Empowerment; DHL, Diabetes Health Literacy; MA, Medication Adherence.

### 3.4 Assessment of common method bias

First, Harman’s single-factor test on the unrotated exploratory factor analysis showed that the first factor explained 32.97% of the total variance, which is below the 40% threshold. Second, we constructed a Common Latent Factor (CLF) model by adding an unmeasured method factor to the baseline measurement model, with all loadings freely estimated and the CLF variance fixed to 1 for identification. Because medication adherence was measured by a single indicator, it was not linked to the CLF to avoid identification problems.

As presented in Table 3, the CLF model exhibited excellent absolute fit (CFI = 0.951, TLI = 0.971, RMSEA = 0.038). Compared with the baseline model, the fit index changes were as follows: ΔCFI = −0.011, ΔTLI = +0.017, and ΔRMSEA = −0.009. The chi-square difference test was significant [Δ*χ*^2^(15) = 41.99, *p <* 0.001], which was not unexpected given the sample size. The absolute change in CFI (|ΔCFI| = 0.011) was marginally above the recommended 0.01 cutoff. However, the baseline model already exhibited excellent fit (CFI = 0.962, RMSEA = 0.047), leaving limited room for the CLF to meaningfully improve model fit. Moreover, the ΔTLI (+0.017) and ΔRMSEA (−0.009) both indicated improved fit, and the absolute fit of the CLF model remained well above all recommended thresholds (CFI > 0.90, TLI > 0.90, RMSEA < 0.05).

**Table 3:** Fit indices comparison for common method bias test.

Model	*χ* ^2^	df	CFI	TLI	RMSEA	Δ*χ*^2^	Δdf	ΔCFI	ΔTLI	ΔRMSEA
Baseline model	156.96	99	0.962	0.954	0.047	–	–	–	–	–
CLF model	114.97	84	0.951	0.971	0.038	41.99	15	−0.011	+0.017	−0.009

In addition, the predictor variables were measured using Likert-type scales, whereas medication adherence was assessed with a different response format, which may reduce the likelihood of artifactual covariation arising from a uniform measurement format. The procedural safeguards taken during the data collection process, such as protecting the anonymity of respondents and emphasizing the absence of right or wrong answers, also help minimize potential methodological biases.

### 3.5 Reliability and validity of the measurement model

As presented in Table 4, the composite reliability (CR) values for all latent constructs ranged from 0.733 to 0.842, all exceeding the recommended threshold of 0.7, indicating satisfactory internal consistency. For discriminant validity, the square root of the AVE for each construct (diagonal entries) exceeded its correlations with the other constructs, meeting the Fornell-Larcker criterion (43).

**Table 4:** Convergent and discriminant validity of the measurement model.

Constructs	CR	Constructs		
		SS	DE	DHL
SS	0.733	**0.696**		
DE	0.769	0.490**	**0.677**	
DHL	0.842	0.623**	0.531**	**0.633**

**p < 0.01; CR, Composite Reliability; Diagonal values in bold represent the square root of AVE. Values in the lower triangle are Pearson correlation coefficients estimated between the latent constructs.

### 3.6 Structural equation model analysis

**Figure 2:**
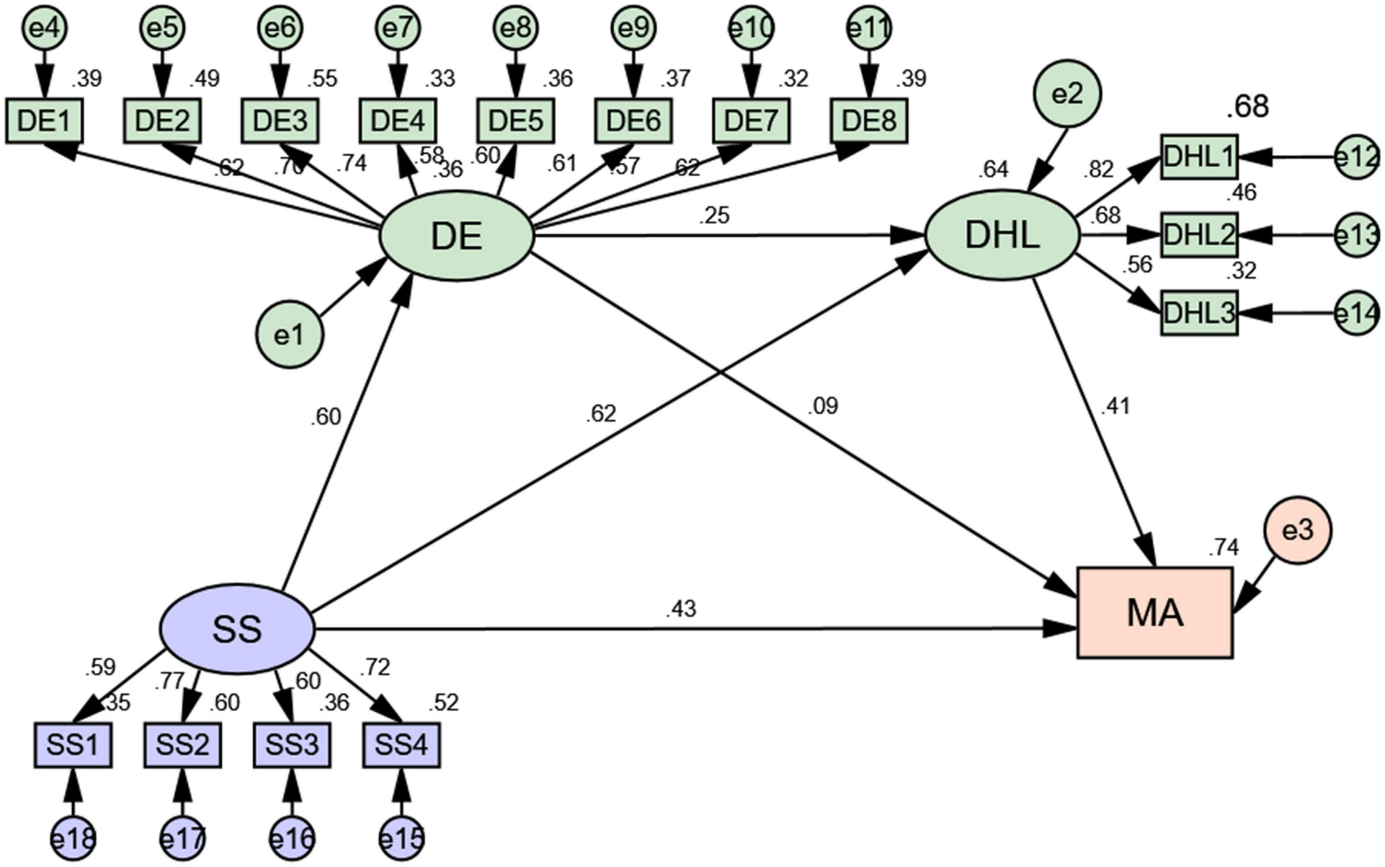
Diagram of the SEM and path coefficient. DE, Diabetes Empowerment; DE1-DE8 refer to the eight items of the Diabetes Empowerment Scale-Short Form (DES-SF); DHL, Diabetes Health Literacy, DHL1-DHL3 refer to the three dimensions of the Diabetes Health Literacy Scale (DHLS): Informational health literacy, Numeracy health literacy, and Communicative health literacy; SS, Social Support, SS1-SS4 refer to the four dimensions of the Medical Outcomes Study Social Support Survey(MOS-SSS-C): Tangible support, Affectionate support, Social interaction support, and Emotional information support; MA, Medication Adherence, it was measured using the Morisky Medication Adherence Scale-8 (MMAS-8). MMAS^®^ 2006 used with permission www.moriskyscale.com.

To test the hypothesized sequential mediation model, we constructed a structural equation model (SEM) with social support as the independent variable, medication adherence as the dependent variable, and diabetes empowerment and diabetes health literacy as the mediating variables. The model demonstrated good fit to the observed data: *χ*^2^/df = 1.585, GFI = 0.933, AGFI = 0.908, CFI = 0.962, RMSEA = 0.047, SRMR = 0.043. All indices met the widely accepted thresholds for acceptable model fit, indicating that the hypothesized model was adequately specified. As shown in Figure 2, all standardized factor loadings of the observed items were statistically significant (*p <* 0.001) and above the conventional threshold of 0.5, supporting satisfactory item-level convergent validity. For the social support construct, the standardized loadings of its four items ranged from 0.59 to 0.77. The indicators for affectionate support (*β* = 0.77) and emotional information support (*β* = 0.72) showed the strongest loadings. The loadings for diabetes empowerment and diabetes health literacy all met the acceptable standard as well.

Figure 2 also presents the standardized path coefficients. Social support showed significant positive direct associations with diabetes empowerment (*β* = 0.60, *p <* 0.001), diabetes health literacy (*β* = 0.62, *p <* 0.001), and medication adherence (*β* = 0.43, *p <* 0.001). Diabetes empowerment was positively associated with health literacy (*β* = 0.25, *p <* 0.01) but was not significantly associated with medication adherence (*β* = 0.09, *p* > 0.05). Diabetes health literacy showed a strong direct association with medication adherence (*β* = 0.41, *p <* 0.001).

### 3.7 Measurement invariance across educational level

To determine whether educational attainment moderates the relationships in the proposed theoretical model, we first tested measurement invariance across groups. A total of 261 patients with T2DM were divided into a low-education group (*n =* 123, junior high school or below) and a high-education group (*n =* 138, senior high school or above). Multi-group structural equation modeling was used to examine configural invariance and metric invariance successively, thereby confirming the measurement equivalence of the scales across the two groups. The results are presented in Table 5.

**Table 5:** Measurement invariance test results across educational level groups.

Model	*χ* ^2^	df	*χ*^2^/df	CFI	RMSEA	Δ*χ*^2^	Δdf	*p*	ΔCFI
Configural invariance	303.72	204	1.49	0.926	0.043	–	–	–	–
Metric invariance	311.45	216	1.44	0.929	0.041	7.73	12	0.806	0.003

Δ*χ*^2^ = Chi-square difference compared with the previous nested model; Δdf = Degrees of freedom difference compared with the previous nested model; p = *p*-value of the chi-square difference test; ΔCFI = Comparative Fit Index difference compared with the previous nested model, a value less than 0.01 indicates invariance holds.

The configural invariance model exhibited good fit (*χ*^2^/df = 1.49 < 3, CFI = 0.926 > 0.9, RMSEA = 0.043 < 0.08), indicating that the factor structure was equivalent across groups. The metric invariance test showed that the model fit did not decrease significantly (Δ*χ*^2^ = 7.73, Δdf = 12, *p* = 0.806; ΔCFI = 0.003 < 0.01), supporting measurement equivalence and ruling out substantial measurement bias in cross-group comparisons. This study further attempted to test structural invariance by constraining the six structural path coefficients to be equal across groups. However, the model failed to converge due to insufficient sample size, thus the differences in structural path strength between the two educational groups were not formally examined in this study.

### 3.8 Test of mediating effects

Table 6 presents the mediating effects of empowerment and health literacy in the relationship between social support and medication adherence. The single mediating effect of empowerment (H2) was not statistically significant (*β* = 0.056, 95% CI [−0.026, 0.147]). In contrast, the single mediating effect of health literacy (H3) was statistically significant (*β* = 0.256, 95% CI [0.133, 0.460]), accounting for 31.7% of the total effect. The sequential mediating effect of empowerment and health literacy (H4) was also significant (*β* = 0.062, 95% CI [0.018, 0.147]), explaining 7.7% of the total effect. The direct effect of social support on medication adherence (H1) was significant (*β* = 0.432, 95% CI [0.182, 0.642]), representing 53.5% of the total effect. The total indirect effect through all mediating pathways was 0.375 (95% CI [0.209, 0.577]), accounting for 46.5% of the total effect, and the overall total effect was 0.807 (95% CI [0.728, 0.871]).

**Table 6:** Mediating effects of empowerment and health literacy.

Hypothesis	Path	Effect value	95% CI	Proportion of effects (%)	Supported
H2	Social support→ empowerment→ medication adherence	0.056	−0.026–0.147	6.9	No
H3	Social support → health literacy → medication adherence	0.256	0.133–0.460	31.7	Yes
H4	Social support→ empowerment→ health literacy→ medication adherence	0.062	0.018–0.147	7.7	Yes
H1	Direct effect (Social support→ medication adherence)	0.432	0.182–0.642	53.5	Yes
	Total indirect effects	0.375	0.209–0.577	46.5	
	Total effect	0.807	0.728–0.871		

Pairwise comparisons of effect magnitudes were conducted using bootstrap confidence intervals to clarify the relative contributions of various pathways connecting social support to medication adherence. Table 7 indicates that the difference between the direct effect of social support on medication adherence (H1, *β* = 0.432) and the single mediating effect of health literacy (H3, *β* = 0.256) was 0.190, but it was not statistically significant (95% CI [−0.276, 0.555], *p* > 0.05). This indicates that these two pathways contributed similarly to the overall association between social support and medication adherence. The direct effect significantly exceeded the sequential mediating effect of empowerment and health literacy (H4, *β* = 0.062), with a difference of 0.398 (95% CI [0.114, 0.712], *p <* 0.05). The single mediating effect of health literacy was also significantly greater than the sequential mediating impact, with a difference of 0.208 (95% CI [0.083, 0.459], *p <* 0.05).

**Table 7:** Pairwise bootstrap comparisons of effect magnitudes.

Pairwise comparison	Effect difference	S. E.	95% CI	*P*
H1 vs. H3	0.190	0.214	−0.276–0.5545	>0.05
H1 vs. H4	0.398	0.156	0.114–0.712	<0.05
H3 vs. H4	0.208	0.094	00.083–0.459	<0.05

### 3.9 Robustness test

After adjusting for age, education level, monthly income and diabetes complications, the overall model fit indices were still acceptable (*χ*^2^/df = 1.567, GFI = 0.921, CFI = 0.955, RMSEA = 0.047, SRMR = 0.045). The model structure remained stable. As shown in Table 8, the direct association between social support and medication adherence was still statistically significant (*β* = 0.486, 95% CI: 0.224–0.699). The single mediating effect of diabetes empowerment remained non-significant after adjustment, with its 95% CI barely including zero, consistent with the primary analysis. For the single mediating pathway of health literacy, the effect estimate decreased upon covariate adjustment and its 95% confidence interval crossed zero. The isolated effect of health literacy partly overlapped with demographic characteristics, particularly educational level, and was not to be interpreted as an independent predictor of adherence in a single-mediator model. The sequential mediating pathway of social support → diabetes empowerment → diabetes health literacy → medication adherence was still supported after adjustment (*β* = 0.033, 95% CI: 0.001–0.114). The effect size decreased slightly but remained statistically significant. These results indicated that the sequential associative mechanism was robust to confounding from the included covariates.

**Table 8:** Standardized mediating effects before and after covariate adjustment.

Path	Unadjusted model Effect value	95% CI	Adjusted model Effect value	95% CI
Social support→ empowerment→ medication adherence	0.056	−0.026–0.147	0.078	0.000–0.168
Social support → health literacy → medication adherence	0.256	0.133–0.460	0.124	−0.002–0.345
Social support→ empowerment→ health literacy→ medication adherence	0.062	0.018–0.147	0.033	0.001–0.114
Direct effect (Social support→ medication adherence)	0.432	0.182–0.642	0.486	0.224–0.699
Total indirect effects	0.375	0.209–0.577	0.235	0.073–0.470
Total effect	0.807	0.728–0.871	0.721	0.637–0.811

Covariates in the adjusted model include age, educational level, monthly income, and diabetes complications. 95% CIs are based on 5,000 bootstrap samples.

## 4 Discussion

This study examined the associations among social support, diabetes empowerment, health literacy, and medication adherence in hospitalized patients with T2DM, and constructed a structural equation model to test the hypothesized pathways. These findings extend existing empirical evidence on multi-path associations with medication adherence, and provide further support for the integrated theoretical model in a hospitalized Chinese T2DM population.

### 4.1 Medication adherence across demographic and clinical characteristics

The study found a moderate level of medication adherence (median *=* 6.75, IQR = 4.50–7.63) among the participants. Medication adherence varied significantly across sociodemographic and clinical subgroups. Female patients, divorced individuals, those aged ≥75 years, those who had an educational attainment of primary school or below, those who had a monthly income ≤3,000 CNY, those who were employed as workers or farmers, and those with diabetes-related complications exhibited relatively lower medication adherence. Among the demographic factors examined, educational level was most strongly associated with medication adherence (H = 67.452, *p <* 0.001). This association may partly reflect the role of health literacy. Patients with less education tend to have lower health literacy, which may in turn be associated with greater challenges in understanding complex treatment plans and disease information, which can adversely affect medication adherence. This interpretation aligns with our subsequent mechanism analysis, which identified health literacy as a proximal mediator, suggesting that the observed educational disparities in adherence likely operate through variations in health literacy.

### 4.2 Direct association between social support and medication adherence

Social support showed a significant positive association with medication adherence, with a direct path coefficient of 0.432 (95% CI [0.182, 0.642]), explaining 53.5% of the total effect. A previous research by Gu et al.(2017) also reported that higher social support was linked to better medication adherence in T2DM patients (44). The current finding is also consistent with the research by Saffari et al. (45), which demonstrated that incorporating social support into interventions was associated with enhanced treatment adherence and the quality of life. In terms of the sub-constructs of social support, the indicators for affectionate support (*β* = 0.77) and emotional information support (*β* = 0.72) showed the highest standardized factor loadings, suggesting that these two dimensions are strong representative indicators of the social support construct in this study population. These findings raise the possibility that interventions emphasizing emotional support and information sharing may warrant further investigation, although causal inferences cannot be drawn from these cross-sectional data.

### 4.3 Mediating pathways of empowerment and health literacy

Empowerment and health literacy jointly mediated the relationship between social support and medication adherence. The robustness test with covariate adjustment further supported the consistency of the observed pattern. The total indirect effect in the model explained 46.5% of the total effect. The single mediating effect of health literacy was significant (*β* = 0.256, 95% CI [0.133, 0.460]), explaining 31.7% of the total effect, highlighting health literacy as a critical intermediary variable. Notably, the single mediating effect of health literacy was attenuated after adjustment. This is consistent with our univariate finding that educational level was the demographic factor most strongly associated with medication adherence. The single mediating effect of empowerment was not significant (*β* = 0.056, 95% CI [−0.026, 0.147]), suggesting that empowerment alone may not be associated with enhanced medication adherence in the absence of a concurrent association with health literacy.

The sequential indirect pathway from social support to medication adherence through empowerment and health literacy was statistically significant (*β* = 0.062, 95% CI [0.018, 0.147]), yet it accounted for only 7.7% of the total effect. After controlling for demographic variables, the sequential mediating pathway was still supported. Although small, this indirect effect suggests a possible internal associative sequence. Greater social support may be associated with stronger empowerment, which may in turn be linked to more active health information seeking, corresponding to higher health literacy and ultimately higher medication adherence. However, the modest magnitude of this pathway (7.7% of the total association) indicates that interventions targeting this sequence alone may have limited practical impact, and future longitudinal research is needed to clarify whether this pathway represents a causal mechanism.

## 5 Limitations and future research

This study has several limitations. First, it was conducted at a single site with convenience sampling, which limits the representativeness of the sample. The findings cannot be readily generalized to all patients with T2DM. Second, the cross-sectional design precludes causal inference and can only reflect associations among variables. The direct consequence of this design is that although the theory we adopted supports the hypothesized sequential pathway of social support → empowerment→ health literacy→ medication adherence, this study cannot exclude the alternative pathway of social support → health literacy → empowerment → medication adherence. The two models are statistically equivalent and cannot be differentiated by fit indices. The true causal ordering among variables therefore requires further clarification through multi-wave longitudinal studies. Third, in this study, data were collected through self-report questionnaires, which may be susceptible to social desirability bias and recall bias. Although we implemented procedural safeguards and both Harman’s single-factor test and a CLF model suggested that common method bias was not a serious threat in this study, the possibility of method effects cannot be entirely ruled out. Fourth, although the overall sample size met the requirements for CB-SEM analysis, the subsample sizes for the low-education (*n =* 123) and high-education (*n =* 138) groups were insufficient to permit a robust multigroup comparison. Consequently, differences in path coefficients across educational levels could not be formally tested.

Future research should seek to address these limitations by adopting a multi-center design with stratified sampling across urban and rural communities in multiple provinces of China, thereby enhancing geographical representativeness and population heterogeneity. A substantially larger sample should be recruited to enable multi-group SEM analysis across educational subgroups. In addition, longitudinal designs incorporating objective adherence monitoring, such as smart pill boxes, wearable devices, are needed to clarify temporal relationships and reduce self-report bias.

## 6 Conclusion

This study tested an integrated associative model of medication adherence in hospitalized patients with T2DM. The results indicate that social support is positively associated with medication adherence, both directly and indirectly, with health literacy serving as an important mediator. A sequential mediating pathway through empowerment and health literacy was also observed, but its magnitude was relatively small (accounting for 7.7% of the total effect). Given the cross-sectional nature of the data, causal inferences cannot be drawn. These findings suggest that strengthening affectionate and emotional information support, as well as improving health literacy, may be promising targets for future longitudinal and interventional research, while the clinical utility of the sequential pathway requires further investigation.

## Data Availability

The original contributions presented in the study are included in the article/Supplementary material, further inquiries can be directed to the corresponding authors.
