# Supplementary material for: Social support and medication adherence in type 2 diabetes: unraveling the sequential mediating pathways of empowerment and health literacy
Source: Front Public Health. 2026 Jul 8;14:1783412. doi: 10.3389/fpubh.2026.1783412 (PMC13388864; doi:10.3389/fpubh.2026.1783412)
Supplement: Supplementary file 2 [file Data_Sheet_2.pdf]

MMAS-8 SCORING GUIDE

Morisky Medication Adherence Scale

Best Practices for Administration, Scoring, and Interpretation

Introduction

The Morisky Medication Adherence Scale (MMAS-8) is a validated self-report instrument for assessing medication-taking behavior. Developed by Dr. Donald E. Morisky, this 8-item scale has been validated in numerous clinical populations and translated into multiple languages through validated studies. The MMAS-8 provides clinicians and researchers with a reliable, standardized method to identify patients at risk for non-adherence and to classify the type of non-adherence to guide targeted interventions.

This guide accompanies the MMAS-8 Self-Assessment Tool and provides detailed instructions for proper administration, scoring methodology, and clinical interpretation of results.

Administering the MMAS-8

Before You Begin

- Ensure the patient understands that responses should reflect their actual medication-taking behavior, not what they believe they should do
- Substitute the specific medical condition and medication name for the placeholder [medical condition] in each question
- Create a non-judgmental environment; patients are more likely to report honestly when they do not fear criticism
- The scale may be self-administered or read aloud by a clinician or researcher

The Eight Questions

Questions 1 through 7 use Yes/No responses. Question 8 uses a 5-point frequency scale.

#	Question	Response	Type
Q1	Do you sometimes forget to take your medicine?	Yes / No	Unintent.
Q2	People sometimes miss taking their medications for reasons other than forgetting. Thinking over the past 2 weeks, were there any times when you did not take your [medical condition] medication?	Yes / No	Intentional
Q3	Would you ever cut back or stop taking your [medical condition] medication without telling your doctor because you felt worse when you took it?	Yes / No	Intentional
Q4	When you travel or leave home, do you sometimes forget to bring along your [medical condition] medication?	Yes / No	Unintent.
Q5	Did you take your [medical condition] medication the last time you were scheduled to take it?	Yes / No	Neutral
Q6	When you feel that your [medical condition] is under control, do you sometimes stop taking your medication?	Yes / No	Intentional
Q7	Taking medication exactly as prescribed is a real inconvenience for some people. Do you ever feel hassled about sticking to your [medical condition] treatment plan?	Yes / No	Intentional
Q8	How often do you have difficulty remembering to take all of your medications?	5-point scale	Unintent.

Scoring Methodology

Questions 1-7: Binary Scoring

For questions 1-4 and 6-7, responses indicating non-adherence receive a score of 0, while responses indicating adherence receive a score of 1.

Question	Yes	No
Q1, Q2, Q3, Q4, Q6, Q7	0 (non-adherent)	1 (adherent)

Question 5: Reverse Scoring

Question 5 is reverse-scored because a "Yes" response indicates the patient DID take their medication (adherent behavior).

Question	Yes	No
Q5	1 (adherent)	0 (non-adherent)

Question 8: Frequency Scale

Question 8 uses a 5-point Likert scale measuring the frequency of difficulty remembering medications:

Response	Score	Interpretation
Never	1.00	Adherent
Rarely	0.75	Mostly adherent
Sometimes	0.50	Partial
Usually	0.25	Limited
All of the time	0.00	Non-adherent

Calculating the Total Score

Sum the scores from all eight questions. The total MMAS-8 score ranges from 0 to 8, with higher scores indicating better medication adherence.

Score Range	Adherence Level	Clinical Interpretation
= 8	**HIGH**	Excellent medication-taking behavior; patient demonstrates full adherence to the prescribed regimen
6 to < 8	**MEDIUM**	Generally good adherence with occasional lapses; monitor progress and address specific barriers
< 6	**LOW**	Significant non-adherence requiring comprehensive assessment and targeted intervention

Understanding Non-Adherence Types

One of the unique strengths of the MMAS-8 is its ability to differentiate between intentional and unintentional non-adherence. This distinction is critical for designing effective interventions, as the underlying causes and appropriate responses differ significantly.

Unintentional Non-Adherence

Assessed by: Questions 1, 4, and 8

Unintentional non-adherence occurs when patients want to take their medications but fail to do so due to forgetfulness, carelessness, or circumstantial barriers. These patients typically have positive attitudes toward their treatment but struggle with the practical aspects of medication-taking.

Common Causes:

- Memory difficulties or cognitive impairment
- Complex medication regimens
- Disruptions to daily routine (travel, schedule changes)
- Lack of organizational systems

Recommended Interventions:

- Pill organizers and medication management systems
- Electronic reminders (smartphone apps, alarm systems)
- Regimen simplification (once-daily dosing, combination products)
- Routine integration (linking medication to daily habits)

Intentional Non-Adherence

Assessed by: Questions 2, 3, 6, and 7

Intentional non-adherence represents a deliberate decision by the patient to deviate from the prescribed regimen. This may stem from beliefs about the medication, concerns about side effects, or perceptions about disease severity. These patients often engage in cost-benefit analyses and conclude that taking medication is not worthwhile.

Common Causes:

- Concerns about side effects or long-term consequences
- Belief that medication is unnecessary (especially when asymptomatic)
- Perception of treatment as burdensome or inconvenient
- Distrust of healthcare providers or the healthcare system

Recommended Interventions:

- Motivational interviewing to explore and address concerns
- Patient education about disease progression and treatment benefits
- Shared decision-making to increase treatment ownership
- Side effect management and medication adjustment when appropriate

Worked Example

Consider a patient with the following responses:

#	Question Summary	Response	Score	Type
Q1	Forget to take medicine?	Yes	0	Unintent.
Q2	Miss for reasons other than forgetting?	No	1	—
Q3	Cut back due to feeling worse?	Yes	0	Intentional
Q4	Forget when traveling?	Yes	0	Unintent.
Q5	Took medication last time?	No	0	Neutral
Q6	Stop when feeling better?	No	1	—

Q7	Feel hassled by treatment?	No	1	—
Q8	Difficulty remembering?	Sometimes	0.5	Unintent.

Total Score: 0 + 1 + 0 + 0 + 0 + 1 + 1 + 0.5 = **3.5**

Adherence Level: LOW (score < 6)

Non-Adherence Pattern: Primarily UNINTENTIONAL (3 unintentional flags vs. 1 intentional)

Recommended Focus: This patient would benefit from memory aids, routine integration strategies, and travel preparation systems. The single intentional flag (Q3) suggests side effects should also be assessed and addressed.

Citation Requirements

Use of the MMAS-8 requires proper attribution. The following citation must be included in all publications, presentations, and reports:

> *Krousel-Wood M, Islam T, Webber LS, Re RN, Morisky DE, Muntner P. New medication adherence scale versus pharmacy fill rates in seniors with hypertension. Am J Manag Care. 2009 Jan;15(1):59-66. PMID: 19146365; PMCID: PMC2728593.*

The following acknowledgment must appear in manuscripts and reports:

> **MMAS® 2006 used with permission www.moriskyscale.com**

Licensing and Support

The MMAS-8 is protected by U.S. copyright laws. Permission is required for use in research, clinical practice, and commercial applications. For licensing inquiries, translation requests, or technical support with the MMAS-8 Self-Assessment Tool:

Morisky Medication Adherence Research, LLC.

www.moriskyscale.com
