# Supplementary material for: Social support and medication adherence in type 2 diabetes: unraveling the sequential mediating pathways of empowerment and health literacy
Source: Front Public Health. 2026 Jul 8;14:1783412. doi: 10.3389/fpubh.2026.1783412 (PMC13388864; doi:10.3389/fpubh.2026.1783412)
Supplement: Supplementary file 3 [file Table_1.DOCX]

Social Support and Medication Adherence in Type 2 Diabetes: Unraveling the Sequential Mediating Pathways of Empowerment and Health Literacy

QN:▢▢▢▢

Dear Patients,

We are researchers from the "Study on the Mechanism of Self-Management Behavior in Diabetic Patients" Research Group at Guizhou Medical University. This study aims to identify the problems diabetic patients encounter in self-management and explore solutions through a questionnaire survey.

We promise that the data obtained in this study will only be used for scientific research and will not be disclosed to the public. Additionally, this is an anonymous questionnaire, so please feel free to fill it out truthfully.

Thank you again for your support!

**Part 1 Demographics**

**1. Your Gender? (Single-choice question)**

A Male　　B Female

**2. Your Age ____ years old (Fill-in-the-blank question)**

**3. Your Educational Background? (Single-choice question)**

A Primary school or below

B Junior high school

C Senior high school/Technical secondary school/Technical school

D associate degree

E Bachelor's degree

F Master's degree or above

**4. Your Monthly Income? (Single-choice question)**

A ≤ 3000 RMB

B 3001 - 5000 RMB

C 5001 - 8000 RMB

D > 8000 RMB

**5. Your Occupation? (Single-choice question)**

A Employees of enterprises, public institutions, and government organizations

B Workers and farmers

C Self-employed individuals

D Retirees

E Others

**6. How many years have passed since you were first diagnosed with diabetes? ____ years (Fill-in-the-blank question)**

**7. Are there any complications? ___**

**Please read each of the following items and mark √ in the space that most closely indicates how much you agree with it (note that there is no correct answer for any of the items).**

**Part 2 Diabetes Health Literacy Scale（DHLS）**

| **Item** | | **Not really** | **Slightly** | **Moderately** | **Quite a lot** | **Very much** |
| --- | --- | --- | --- | --- | --- | --- |
| A1 | I can read and understand the educational materials and booklets on diabetes. |  |  |  |  |  |
| A2 | I understand the written information provided at an appointment for diabetes treatment or an examination. |  |  |  |  |  |
| A3 | I comprehend the information I sought on diabetes. |  |  |  |  |  |
| A4 | I understand the information on diabetes management provided by the health-care provider. |  |  |  |  |  |
| A5 | I can judge if diabetes-related information is reliable. |  |  |  |  |  |
| A6 | I can print out my prescription from an automated prescription machine at the hospital. |  |  |  |  |  |
| A7 | When a change occurs in my personal schedule, I can alter the appointment date or time for a medical checkup. |  |  |  |  |  |
| A8 | I can calculate the next time to take diabetes medication. |  |  |  |  |  |
| A9 | I can determine the carbohydrate content per serving from the nutrition label on food packaging. |  |  |  |  |  |
| A10 | I can interpret if my blood-glucose level is within the normal range. |  |  |  |  |  |
| A11 | I can understand information on diabetes presented as probabilities, ratios, or on graphs. |  |  |  |  |  |
| A12 | When I have a question about diabetes, I usually ask a health-care provider. |  |  |  |  |  |
| A13 | I can explain my diabetes condition to a health-care provider. |  |  |  |  |  |
| A14 | When eating out with colleagues or friends, I can convey the reason why I should have a diabetic diet. |  |  |  |  |  |

**Part 3 Social Support Scale** **（MOS-SSS）**

| **Item** | | **none of the time** | **A little of the time** | **Some of the time** | **Most of the time** | **all of the time** |
| --- | --- | --- | --- | --- | --- | --- |
| B1 | Someone to help if you were confined in bed |  |  |  |  |  |
| B2 | Someone to confide in or talk to about yourself or your problems |  |  |  |  |  |
| B3 | Someone to give you good advice about a crisis |  |  |  |  |  |
| B4 | Someone to take you to the doctor if you needed it |  |  |  |  |  |
| B5 | Someone who shows you love and affection |  |  |  |  |  |
| B6 | Someone to have a good time with |  |  |  |  |  |
| B7 | Someone to give you information to help you understand a situation |  |  |  |  |  |
| B8 | Someone you can count on to listen to you when you need to talk |  |  |  |  |  |
| B9 | Someone who hugs you |  |  |  |  |  |
| B10 | Someone to get together with for relaxation |  |  |  |  |  |
| B11 | Someone to prepare your meals if you were unable to do it yourself |  |  |  |  |  |
| B12 | Someone whose advice you really want |  |  |  |  |  |
| B13 | Someone to do things with to help you get your mind off things |  |  |  |  |  |
| B14 | Someone to help with daily chores if you were sick |  |  |  |  |  |
| B15 | Someone to share your most private worries and fears with |  |  |  |  |  |
| B16 | Someone to turn to for suggestions about how to deal with a personal problem |  |  |  |  |  |
| B17 | Someone to do something enjoyable with |  |  |  |  |  |
| B18 | Someone who understands your problems |  |  |  |  |  |
| B19 | Someone who love you and makes you feel wanted |  |  |  |  |  |

**Part 4 The Diabetes Empowerment Scale-Short Form (DES-SF)**

| Item | | Strongly disagree | Somewhat disagree | neutral | Somewhat agree | Strongly agree |
| --- | --- | --- | --- | --- | --- | --- |
| C1 | I know what parts of taking care of my diabetes that I am dissatisfied with. |  |  |  |  |  |
| C2 | I am able to turn my diabetes goals into a workable plan. |  |  |  |  |  |
| C3 | I can try out different ways of overcoming barriers to my diabetes goals. |  |  |  |  |  |
| C4 | I can find ways to feel better about having diabetes. |  |  |  |  |  |
| C5 | I know the positive ways I cope with diabetes-related stress. |  |  |  |  |  |
| C6 | I can ask for support for having and caring for my diabetes when I need it. |  |  |  |  |  |
| C7 | I know what helps me stay motivated to care for my diabetes. |  |  |  |  |  |
| C8 | I know enough about myself as a person to make diabetes care choices that are right for me. |  |  |  |  |  |

**Part 5 8-item Morisky Medication Adherence Scale (MMAS-8)**

| Item | | Yes | No |
| --- | --- | --- | --- |
| D1 | Do you sometimes forget to take your medicine? |  |  |
| D2 | People sometimes miss taking their medications for reasons other than forgetting. Thinking over the past 2 weeks, were there any times when you did not take your type 2 diabetes medication? |  |  |
| D3 | Would you ever cut back or stop taking your type 2 diabetes medication without telling your doctor because you felt worse when you took it? |  |  |
| D4 | When you travel or leave home, do you sometimes forget to bring along your type 2 diabetes medication? |  |  |
| D5 | Did you take your type 2 diabetes medication the last time you were scheduled to take it? |  |  |
| D6 | When you feel that your type 2 diabetes is under control, do you sometimes stop taking your medication? |  |  |
| D7 | Taking medication exactly as prescribed is a real inconvenience for some people. Do you ever feel hassled about sticking to your type 2 diabetes  treatment plan? |  |  |
| D8 | How often do you have difficulty remembering to take all of your medications? | Never/Rarely；Once in a while；Sometimes；Usually；All the time | |
